# Enzymatic Synthesis and Antimicrobial Activity of Oligomer Analogues of Medicinal Biopolymers from Comfrey and Other Species of the Boraginaceae Family

**DOI:** 10.3390/pharmaceutics14010115

**Published:** 2022-01-04

**Authors:** Maia Merlani, Dieter M. Scheibel, Vakhtang Barbakadze, Lali Gogilashvili, Lela Amiranashvili, Athina Geronikaki, Valentina Catania, Domenico Schillaci, Giuseppe Gallo, Ivan Gitsov

**Affiliations:** 1 Kutateladze Institute of Pharmacochemistry, Tbilisi State Medical University, 36 P.Sarajishvili Street, 0159 Tbilisi, Georgia; m.merlani@tsmu.edu (M.M.); v.barbakadze@tsmu.edu (V.B.); l.gogilashvili@tsmu.edu (L.G.); l.amiranashvili@tsmu.edu (L.A.); 2 Department of Chemistry, College of Environmental Science and Forestry, State University of New York, 1 Forestry Drive, Syracuse, NY 13210, USA; dmscheib@syr.edu; 3 School of Pharmacy, Aristotle University, 54124 Thessaloniki, Greece; geronik@pharm.auth.gr; 4 Laboratory of Microbiology and Biologic Assays, Department of Biological, Chemical and Pharmaceutical Sciences and Technologies, University of Palermo, 32 Via Archirafi, 90123 Palermo, Italy; valentina.catania@unipa.it (V.C.); domenico.schillaci@unipa.it (D.S.); 5 Laboratory of Molecular Microbiology and Biotechnology, Department of Biological, Chemical and Pharmaceutical Sciences and Technologies, University of Palermo, Viale delle Scienze, ed. 16, 90128 Palermo, Italy; giuseppe.gallo@unipa.it; 6 The Michael M. Szwarc Polymer Research Institute, Syracuse, NY 13210, USA; 7 The BioInspired Institute, Syracuse University, Syracuse, NY 13210, USA

**Keywords:** poly[3-(3,4-dihydroxyphenyl)glyceric acid], enzymatic polymerization, lipase, antimicrobial activity, Boraginaceae family

## Abstract

This study reports the first enzymatic synthesis leading to several oligomer analogues of poly[3-(3,4-dihydroxyphenyl)glyceric acid]. This biopolymer, extracted from plants of the Boraginaceae family has shown a wide spectrum of pharmacological properties, including antimicrobial activity. Enzymatic ring opening polymerization of 2-methoxycarbonyl-3-(3,4-dibenzyloxyphenyl)oxirane (MDBPO) using lipase from *Candida rugosa* leads to formation of poly[2-methoxycarbonyl-3-(3,4-dibenzyloxyphenyl)oxirane] (PMDBPO), with a degree of polymerization up to 5. Catalytic debenzylation of PMDBPO using H_2_ on Pd/C yields poly[2-methoxycarbonyl-3-(3,4-dihydroxyphenyl)oxirane] (PMDHPO) without loss in molecular mass. Antibacterial assessment of natural polyethers from different species of Boraginaceae family *Symhytum asperum*, *S. caucasicum,* *S. grandiflorum*, *Anchusa italica*, *Cynoglossum officinale,* and synthetic polymers, poly[2-methoxycarbonyl-3-(3,4-dimethoxyphenyl)oxirane (PMDMPO) and PMDHPO, reveals that only the synthetic analogue produced in this study (PMDHPO) exhibits a promising antimicrobial activity against pathogenic strains *S.aureus* ATCC 25923 and *E.coli* ATCC 25922 the minimum inhibitory concentration (MIC) being 100 µg/mL.

## 1. Introduction

**Figure 1:**
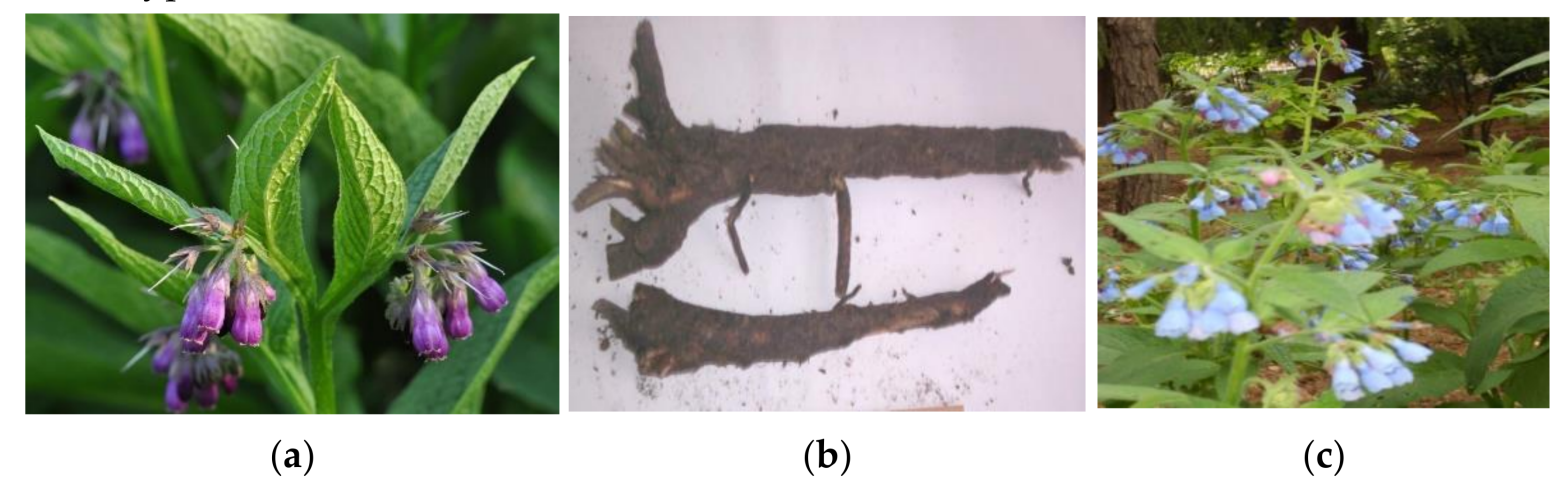
(**a**) Comfrey (*S. officinale*) plant leaves and flower. This file is licensed under the Creative Commons Attribution-Share Alike 3.0 Unported. credit to: User: Aiwok/Wikimedia Commons/CC-BY-SA-3.0 [6] (**b**) *A. Italica* (roots). Source: A voucher specimen TBPH-224 of *A.italica* is deposited in the Herbarium of the Institute of Pharmacochemistry, Tbilisi State Medical University (TSMU), Tbilisi, Georgia. (**c**) *S. caucasicum.* Source: A voucher specimen TBPH-20996 of *S. caucasicum* is deposited in the Herbarium of the Institute of Pharmacochemistry, TSMU, Tbilisi, Georgia.

Several plants from the Boraginaceae family such as *Symphytum officinale L.* (*S. officinale,* Figure 1a), comfrey, and *Anchusa italica (A. italica)* synonymous for *Anchusa azurea* (*A.azurea*) are known to exhibit a broad pharmacological activity [1,2,3,4,5]. Comfrey was used in traditional medicine for bone breakages, sprains and rheumatism, liver problems, gastritis, ulcers, skin disorders, joint pain and contusions, wounds, gout, hematomas, and thrombophlebitis [1], while *A. italica*-as stimulant, tonic, demulcent, in bilious complaints, fever, cough, asthma and as diuretic in bladder and kidney stones, as diaphoretic, narcotic, hypnotic, antiarthritis, antirheumatic, and cathartic [4].

Extracts of comfrey and *A. italica* showed potential antimicrobial effects on several bacterial strains tested, especially against *Staphylococcus aureus* ATCC 25923 (*S. aureus*) [1,5], as well as antifungal activity against *Bipolaris oryzae* (*B.oryzae*) (comfrey) [1]. It was highlighted that the effect of the investigated extracts could be attributed to a mix of components including phenolic compounds (caffeic and chlorogenic acids, luteolin glycoside) and allantoin, possibly due to the synergy between compounds, as well as with other bioactive molecules present in the whole plants [1,5]. Since recent research revealed that allantoin is not responsible for the antimicrobial activity of the aqueous extract of comfrey root (Figure 1b) [7], preclinical studies regarding antimicrobial activities of comfrey extracts [1] demonstrates the need for deep investigations of extracts from all parts of plants, isolation, and chemical structure determination of bioactive compounds.

**Figure 2:**
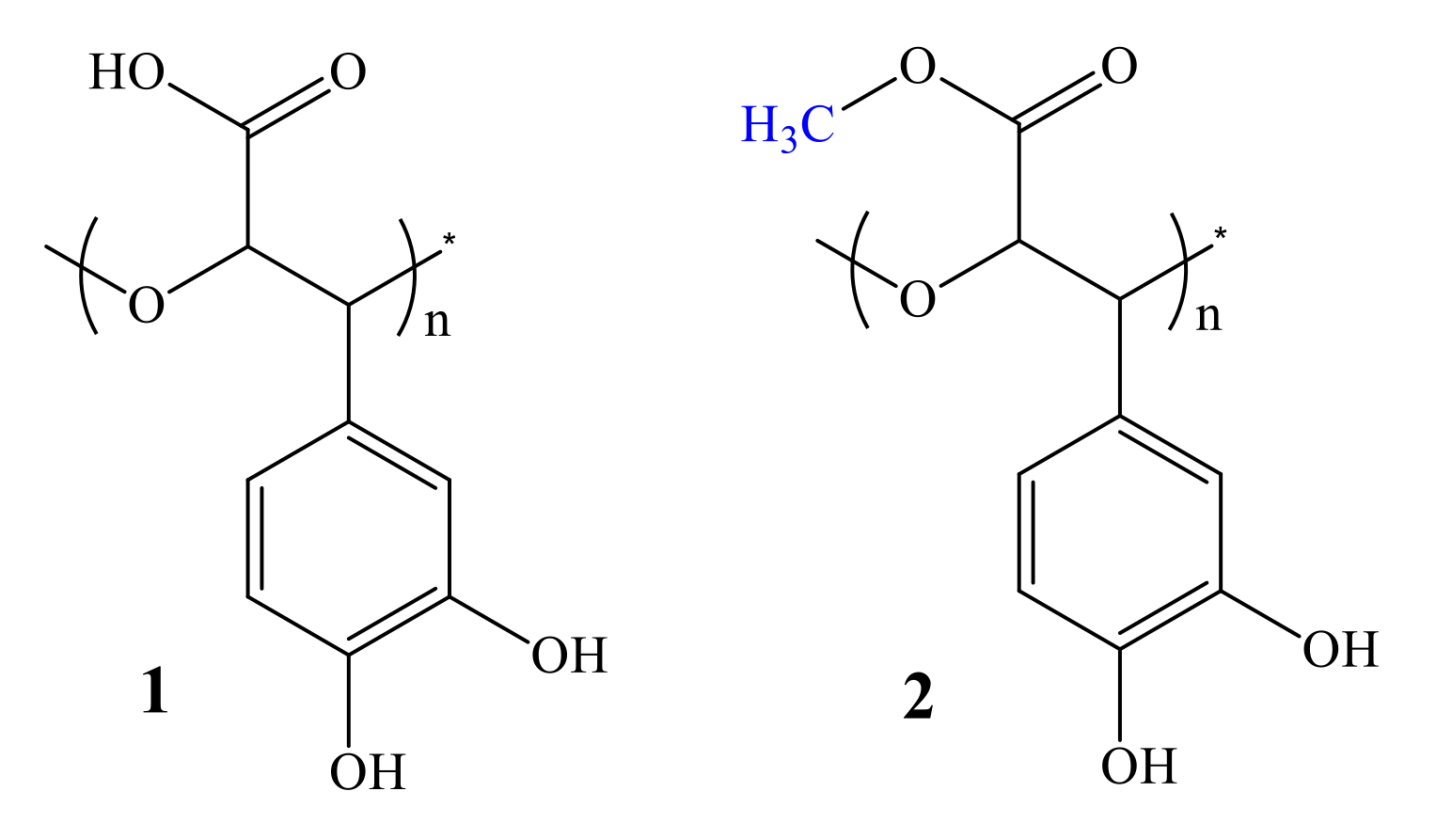
Poly[3-(3,4-dihydroxyphenyl)glyceric acid] (PDHPGA) (**1**) and poly[2-methoxycarbonyl-3-(3,4-dihydroxyphenyl)oxirane] (PMDHPO) (**2**).

In our previous study poly [3-(3,4-dihydroxyphenyl)glyceric acid] (PDHPGA) (Figure 2(**1**)), was isolated within high molecular fractions (HMF) from the roots and stems of different species of Boraginaceae family: *S. officinale* (SO), *Symphytum asperum* (SA), *Symphytum caucasicum* (Figure 1c, SC), *S. grandiflorum* (SG), *A. italica* (AI), *Cynoglossum officinale* (CO), and *Borago officinalis* (BO). The repeating unit of PDHPGA is 3-(3,4-dihydroxyphenyl)glyceric acid residue [8,9,10,11,12,13,14].

Distinguished from PDHPGA-SA, PDHPGA-SC, PDHPGA–SO, and PDHPGA–CO most of the carboxylic groups of PDHPGA-SG, PDHPGA-AI, and PDHPGA-BO are methylated [11,12,14], (Figure 2(**2**)). Water soluble PDHPGA exhibits a wide spectrum of pharmacological activities: immunomodulatory (anticomplementary), antioxidative, anti-inflammatory, wound and burn healing, and anticancer both in vitro and in vivo [15,16,17,18,19,20]. The synthesized monomer of the natural polymer: 3-(3,4-dihydroxyphenyl)glyceric acid showed high anticancer [20] and antioxidant activities [21] in both racemic and enantiomeric forms. High antimicrobial and antifungal activities were reported by some newly synthesized derivatives of caffeic and 3-(3,4-dihydroxyphenyl)glyceric acids, as well [22]. Medicinal effects of *Symphytum* and *Anchusa* plants used in folk medicine could be attributed not just to some low molecular mass phenolic compounds and the abovementioned bioactive molecules but to PDHPGA as well. PDHPGA is a unique natural polyether, as it contains aliphatic ether groups in its polymer backbone. Thus, PDHPGA and its analogues can be promising candidates for many biomedical applications based on their outstanding bioactive characteristics. The formation of those analogues by avoiding toxic catalysts is a challenging but highly beneficial undertaking, and that is the focus of the current study where the major aims are: a) the preparation of PDHPGA synthetic analogues by using enzyme-mediated polymerization and b) the evaluation of antimicrobial activity of the polymers formed.

## 2. Materials and Methods

### 2.1. Materials

3,4-dibenzyloxybenzaldehyde (98%, Sigma-Aldrich, Milwaukee, WI, USA), methyl chloroacetate (99%, Sigma-Aldrich, USA), CDCl_3_ and acetone-d_6_ (D, 99.8%, Cambridge Isotope Laboratories, Inc. Andover, MA, USA) and dimethyl sulfoxide, DMSO (99.5%, Carlo Erba, Cornaredo, Italy) were used without further purification. Tetrahydrofuran, THF (anhydrous 99.9%), toluene (anhydrous, 99.8%), 2,5-dihydroxybenzoic acid, DHB (98%), lipase from *Candida rugosa* (≥700 U/mg), and Amano lipase G from *Penicillium camemberti* (≥5000 U/mg) were all purchased from Sigma-Aldrich, USA, and were used as received.

#### 2.1.1. 2-Methoxycarbonyl-3-(3,4-Dibenzyloxyphenyl)-Oxirane (MDBPO) (4)

A solution of 3,4-dibenzyloxybenzaldehyde (0.47 g, 1.5 mmol) and methyl chloroacetate (0.24 g, 2.25 mmol) in 3 mL THF was added to a suspension of Na (0.05 g, 2.25 mmol) in dry methanol (1.0 mL) at −10 °C during a period of 30 min. The mixture was stirred at −5 °C for 2 h and then at room temperature for 3 h. The mixture was poured into 10 mL ice water containing acetic acid (0.01 mL). Crude products precipitated as a white powder, which was collected by filtration, washed with water, and dried *in vacuo*. The crude material was recrystallized from diethyl ether to yield glycidate **4** as a white solid, m.p. 59–61 °C. Yield: 0.28 g (50%, th) IR (KBr, cm^−1^) ν = 1750, 1514, 1267, 1245, 1217, 1197, 1161, 1138, 991, 916, 898, 791, 773, 750, and 741; ^1^H NMR (600 MHz, CDCl_3_, TMS, 25 °C), δ 3.44 (d, *J* = 1.7 Hz, 1H), 3.83 (s, 3H), 4.02 (d, *J* = 1.7 Hz, 1H), 5.17(s, 2H), 5,18 (s, 2H), 6.85 (d, *J* = 2.0 Hz, 1H), 6.86 (dd, *J* = 8.03, 2.0 Hz), and 6.93 (d, *J* = 8.0 Hz, 1H) ppm; ^13^C NMR (150 MHz, CDCl_3_, TMS, 25 °C) δ 52.57, 56.579, 58.10, 71.30, 71.52, 112.11, 114.99, 119.53, 127.24–128.53, 136.95, 136.99, 149.32, 149,73, and 168.72 ppm. MALDI TOF MS: m/z = 391.769 (M^+1^).

#### 2.1.2. Poly[2-methoxycarbonyl-3-(3,4-dibenzyloxyphenyl)oxirane] PMDBPO (**7**)

In a typical polymerization, methyl 3-(3,4-dibenzyloxyphenyl)glycidate **4** (50 mg, 0.128 mmol) was dissolved in anhydrous toluene (500 µL). To this solution, lipase from *Candida rugosa* (20 mg) was added forming a suspension. Polymerization was initiated by heating the suspension in an oil bath at 80 °C and was allowed to react for 7 days under vigorous stirring. Polymerization was halted by addition of THF (5 mL) and subsequent filtration through a 0.2 µm PTFE filter to remove the biocatalyst. The product (**7**) was concentrated in a rotary evaporator and obtained as a viscous yellow oil.

#### 2.1.3. Poly[2-methoxycarbonyl-3-(3,4-dihydroxyphenyl)oxirane] PMDHPO (**2**)

A solution of 1.5 g of poly[2-methoxycarbonyl-3-(3,4-dibenzyloxyphenyl)oxirane], **7**, in a 1:1 mixture of ethanol-THF (20 mL) was added to a stirred suspension of palladium/carbon (10 mol%) in the same solvent system (20 mL) that had previously been evacuated, purged with hydrogen, and stirred for 30 min under a hydrogen atmosphere. The reaction mixture was stirred overnight and then filtered through celite. The organic extracts were concentrated under vacuum. Product **2** was obtained as yellowish oil. Yield: 1.193 g (79.53%, th).

### 2.2. Methods

^1^H NMR and ^13^C NMR spectra were recorded using CDCl_3_ or acetone-d_6_ as a solvent at 22 °C with a Bruker AVANCE 600 MHz instrument (Bruker Co., Billerica, MA, USA).

Fourier transform infrared spectroscopy (FT-IR) spectra were obtained on a Bruker Tensor 27 spectrophotometer with an MIR source and a DLaTGS detector all from Bruker Co., USA. Spectra were recorded under ambient conditions at a resolution of 4 cm^−1^. A total of 64 scans were recorded for each spectrum in addition to the background.

Size exclusion chromatography (SEC) was conducted on a system consisting of an M510 pump, a U6K universal injector (both from Waters Co., Milford, MA, USA), three 5 μm PL Gel columns (50 Å, 500 Å, and Mixed C), and a Viscotek 250 dual refractive index/viscometry detector (Malvern Panalytical, Malvern, UK). All analyses were conducted at 45 °C with freshly distilled THF at a flow rate of 0.8 mL/min. Calibration was performed using 20 monodisperse poly(styrene) standards (0.162−1670 kDa, Polymer Standards Service, Amherst, MA, USA) and Viscotek OmniSEC 5.0 software (Malvern Panalytical, UK).

Matrix-assisted laser desorption/ionization-time of flight mass spectrometry (MALDI TOF MS) spectra were acquired on a Bruker Autoflex III (Bruker Co., USA) equipped with Smartbeam II laser source (Nd-YAG laser, 266 or 355 nm). Spectra were collected in linear positive mode with the attenuation set to the lowest value capable of obtaining high resolution spectra. Matrix solution was prepared by dissolving DHB in acetone at a concentration of 80 mg/mL. Sample solutions were prepared in acetone at a concentration of approximately 1 mg/mL. Samples were spotted by mixing matrix solution and sample solution in a 1:1 ratio and spotting 5 µL of the resulting mixture on an MTP 384 target plate (polished steel, Bruker Daltonics, Bruker Co., USA).

#### Determination of Minimum Inhibitory Concentration (MIC) and Minimum Bactericidal Concentration (MBC)

In order to ascertain that the possible antimicrobial activity is due to the effect of polymeric substances, pure DMSO (used to dissolve PMDMPO and PMDBPO polymers) was preliminarily tested alone for antimicrobial activity by growth inhibition assay at the maximal concentration (2% *v*/*v*) used for polymer treatment of bacterial tester cells. The results revealed that pure DMSO does not affect bacterial growth at the concentration assayed.

MICs were determined by a microdilution method [23]. Briefly, a series of polymer solutions (i.e., PDHPGA-SA, PDHPGA-SC, PDHPGA-SG, PDHPGA-AI, PDHPGA-CO, PMDMPO, PMDBPO, and PMDHPO) were prepared with a range of concentrations from 100 to 0.75 µg/mL obtained by two-fold serial dilution [24]. The serial dilutions were made in Mueller–Hinton broth (MH) (Sigma Aldrich) in a 96-well plate, starting from a stock solution of 5 mg/mL in DMSO or water. To each well was added 10 µL of a bacterial suspension, obtained from a 24 h culture grown at 37 °C on Tryptic Soy Agar (TSA), containing ~10^6^ cfu/mL. A positive and negative control, consisting respectively of bacterial strains in the medium without extract, and the medium without both extract and inoculum were also included in the 96-well plate. A substance control, consisting only of the substance solution without bacterial inoculum, was added to evaluate the absorbance of the substance. The plate was incubated at 37 °C for 24 h. The MIC was determined by a microplate reader (Glomax®-Multi Detection System TM297, Promega, Milano, Italy) as the lowest concentration of compound whose optical density (OD) at 570 nm, was comparable with the negative control wells (broth only without inoculum). To determine the MBC, 100 μL of subculture from each negative well and from the positive control of MIC determination was put onto a TSA plate for 24 h at 37 °C. The MBC is defined as the lowest concentration of substance allowing microbial growth up to a maximum of three colonies.

## 3. Results and Discussion

Many biocatalytic polymerizations have been exploited for the synthesis of polymers in vitro [25,26,27] including some recent research from our group [28,29,30]. Furthermore, the biocatalytic synthesis of ethers in vitro and in vivo is also known [31,32]. Naturally occurring ethers include small molecules such as antibiotics, or aromatic polymers such as lignin. In the latter case, peroxidases initiate the radical coupling of monolignols to lignin, yielding ether links between two aromatic rings or between an aromatic ring and an aliphatic moiety [32]. However, reports on the enzymatic synthesis of polymers that contain aliphatic ethers as repeating unit are sparse. These include the ring-opening-polymerization (ROP) of glycidol by lipases which yielded linear poly(glycidol) [33,34,35]. Additionally, lipase-catalyzed co-polymerizations of glycidol and acid anhydrides have been reported [34,35,36]. On the other hand, the biosynthesis of the rather rare class of natural poly(ether)s, that PDHPGA belongs to, has not been attempted.

**Figure 3:**
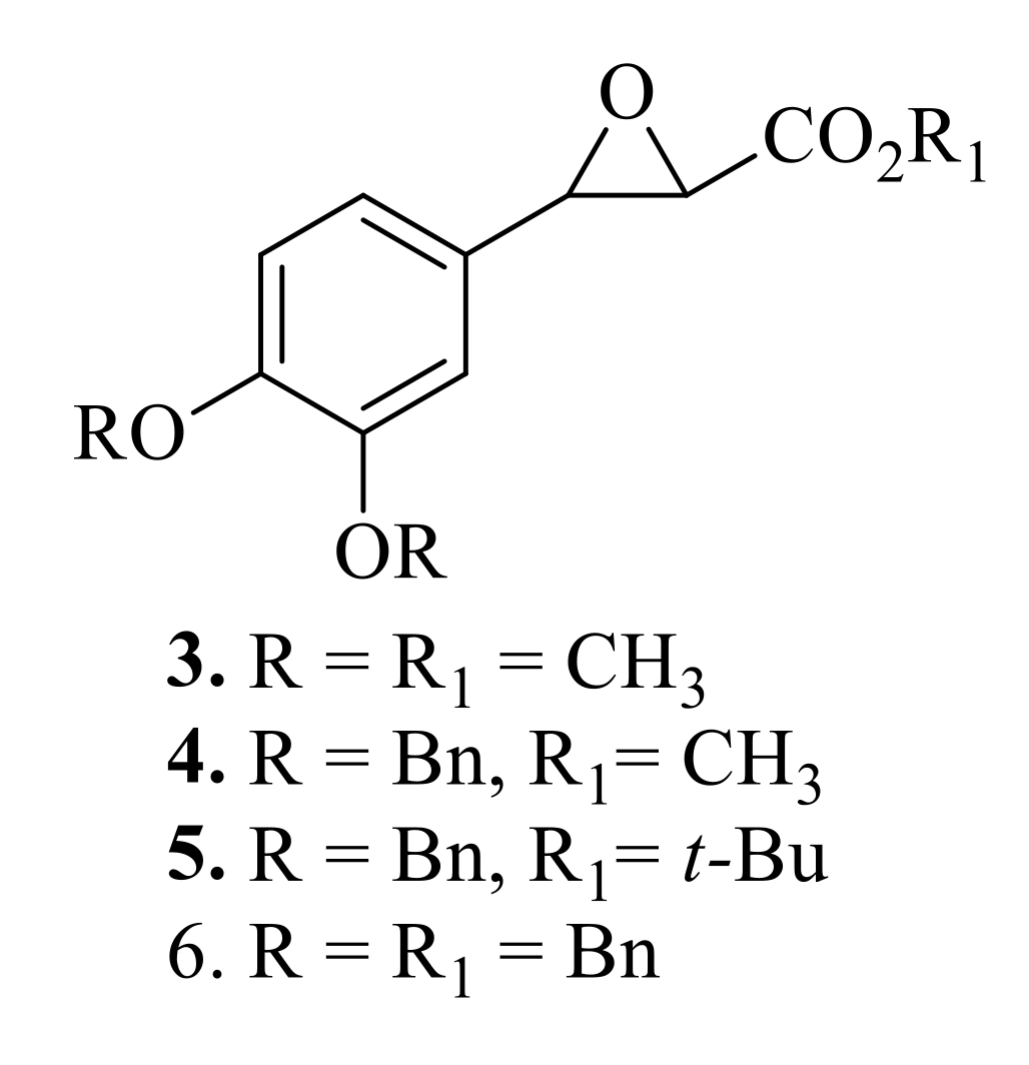
2-methoxycarbonyl-3-(3,4-dimethoxyphenyl)oxirane (MDMPO) (**3**), 2-methoxycarbonyl-3-(3,4-dibenzyloxyphenyl)-oxirane (MDBPO) (**4**), 2-benzyloxycarbonyl-3-(3,4-dibenzyloxyphenyl)oxirane (BDBPO) (**5**), 2-*t*-butyloxycarbonyl-3-(3,4-dibenzyloxyphenyl)oxirane (TBDBPO) (**6**).

We have recently reported the synthesis and cationic polymerization of 2-methoxycarbonyl-3-(3,4-dimethoxyphenyl)oxirane (MDMPO) (Figure 3(**3**)) [37], 2-methoxy-3-(3,4-dibenzyloxyphenyl)-oxirane (MDBPO) (Figure 3(**4**)), 2-benzyloxycarbonyl-3-(3,4-dibenzyloxyphenyl)oxirane (BDBPO) (Figure 3(**5**)), and 2-*t*-butyloxycarbonyl-3-(3,4-dibenzyloxyphenyl)oxirane (TBDBPO) (Figure 3(**6**)) [38]. The molecular masses of the obtained polymers poly(MDBPO) and poly(DBBPO) (*M_n_* = 810–2755 Da and 970–2840 Da, respectively) were markedly lower than those of poly(MDMPO)-*M_n_* 3800–12 800 Da (vs polystyrene standards). TBDBPO did not polymerize under various conditions. These results might have been caused by the presence of bulky substituents in MDBPO, DBBPO, and TBDBPO, compared with MDMPO [38].

### 3.1. Enzymatic Polymerization

**Scheme 1:**
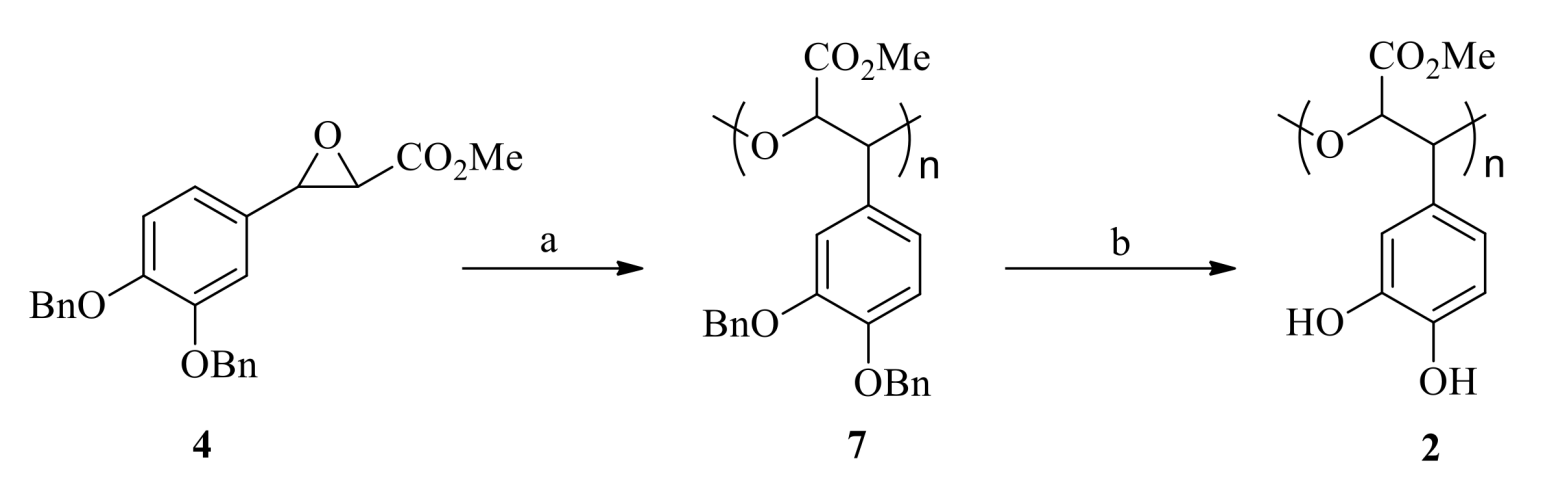
Enzymatic polymerization of MDBPO and modification of the polymer: (**a**) *C. rugosa* lipase, toluene, 80 °C, 7 days (**b**) Pd/C, H_2_, THF/EtOH.

**Figure 4:**
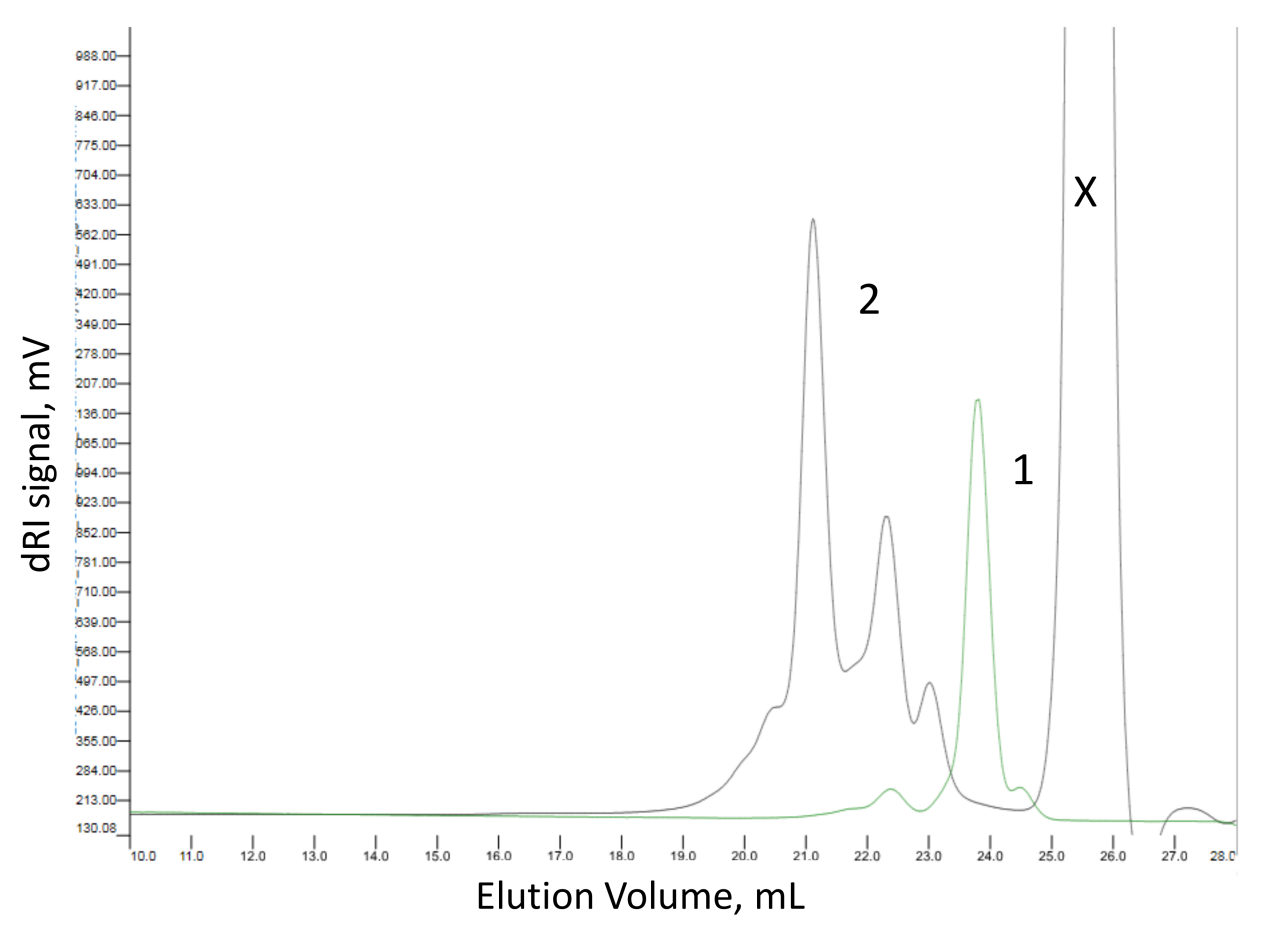
SEC traces of MDBPO monomer (1, green trace) and polymer PMDBPO (2, black trace). X—internal standard. See experimental for analysis conditions.

**Figure 5:**
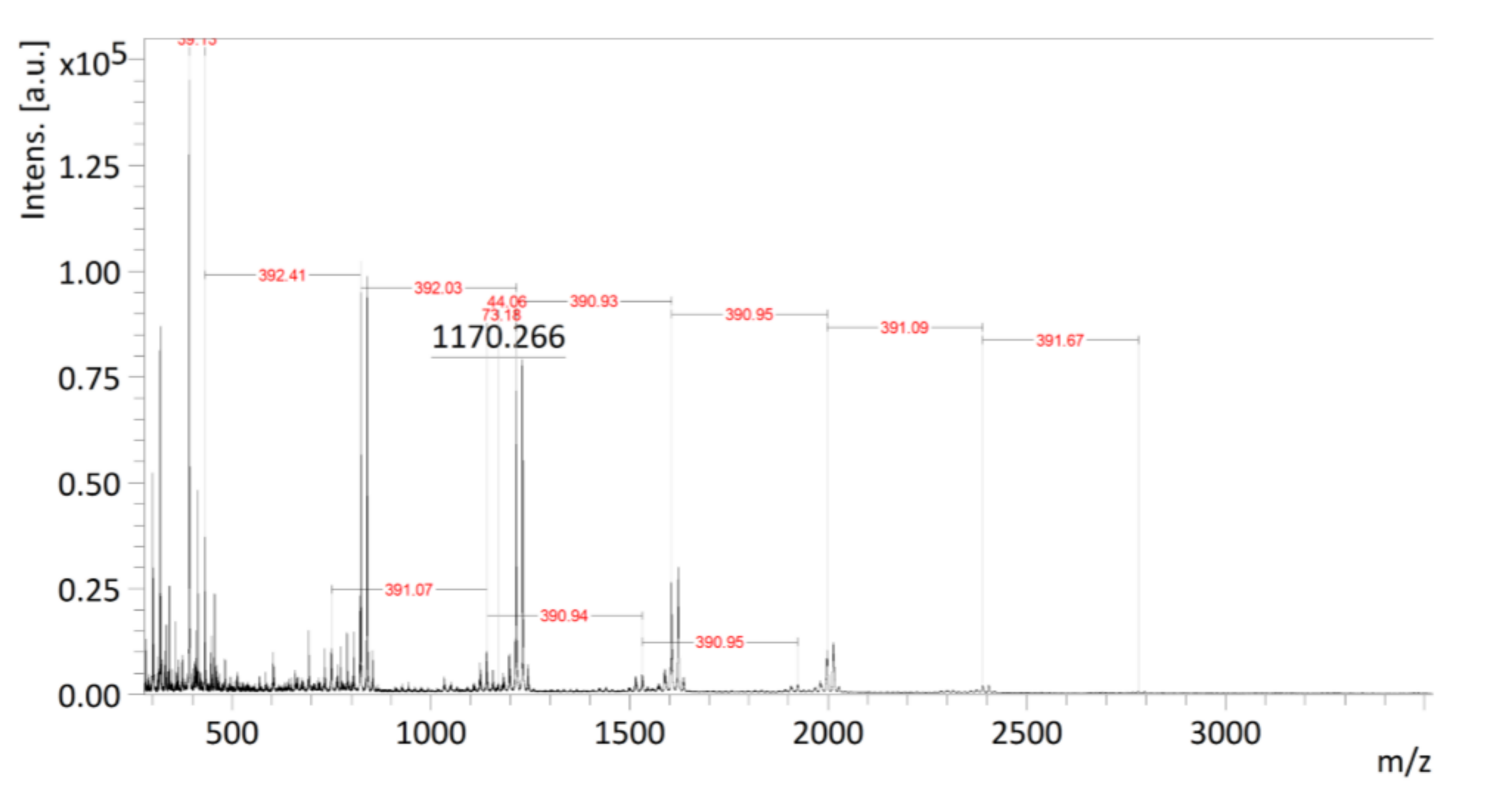
MALDI-TOF mass spectrum of PMDBPO. See experimental for analysis conditions.

In this study, we attempted the polymerization of glycidates (**4–6**) under mild and more environmentally-friendly conditions using lipases from two different sources-*Candida rugosa* (*C. rugosa*) and *Penicillium camemberti* (*P. camemberti*). In our previous study [28], they showed high catalytic activity towards various substrates. The polymerization was carried out in anhydrous toluene at 80 °C for 7 days using a biocatalyst concentration of 4%, Scheme 1a. We also found lipase from *C. rugosa* was the most efficient in inducing the ring-opening polymerization of MDBPO while the enzyme from *P. camemberti* was not active under the polymerization conditions chosen. SEC (Figure 4) and MALDI-TOF analyses (Figure 5) showed the formation of poly(MDBPO) (**7**) with a degree of polymerization up to 5. The mass spectrum contained well resolved signals spaced with mass numbers, which were close to the molar mass of the expected repeating unit (390.1); the observed mass numbers of each individual peak roughly corresponded to molar masses of polymer structures with a –OH group at the terminal (**7**). Due to the nature of this analysis, the existence of higher oligomers could not be confirmed or excluded. The reactions with BDBPO and TBDBPO did not show any trace of polymer, most probably due to the steric hindrance by the benzyloxy- and *t*-butyloxy-groups. Catalytic debenzylation of PMDBPO (**7**) using H_2_ on Pd/C yielded the synthetic analogue of poly(MDHPO) (**2**) at ~80% yield and without loss in molecular mass (Scheme 1b).

### 3.2. Antimicrobial Activity

The substances to be tested by microbiological assay were initially solubilized in distilled water or DMSO (5 mg/mL) and were sterilized by microfiltration.

The samples were subjected to an evaluation of antimicrobial activity against the following important human pathogenic bacterial strains: *Staphylococcus aureus* ATCC 25923, *Pseudomonas aeruginosa* ATCC 15442, *Enterococcus faecalis* ATCC 29212, *Escherichia coli* ATCC 25922. Antimicrobial activity against free-living strains (i.e., planktonic cell cultivation) was evaluated using Muller-Hinton broth (MHB) growth medium and was expressed in terms of minimum inhibitory concentration (MIC). For each substance, the antimicrobial activity was assessed at a range of concentrations from 100 to 0.75 µg/mL (obtained by two-fold serial dilution). Among the polymers evaluated (PDHPGA-SA, PDHPGA-SC, PDHPGA-SG, PDHPGA-AI, PDHPGA-CO, PMDMPO, PMDBPO, and PMDHPO) only PMDHPO showed antimicrobial activity at the tested concentration, Table 1. In particular, PMDHPO was active against *S. aureus* ATCC 25923 and *E. coli* ATCC 25922 in MHB growth medium at the maximum concentration used. Interestingly, the MICs against *S. aureus* ATCC 25923 and *E. coli* ATCC 25922 also coincided with the minimum bactericidal concentration (MBC) of the PMDHPO as it was revealed by counting colony forming units (CFU) of bacterial cultures obtained by plating aliquots of treated cultivations on TSA growth medium.

## 4. Conclusions

This is the first attempt to produce a synthetic analogue of natural polymer poly[2-methoxycarbonyl-3-(3,4-dihydroxyphenyl)oxirane], isolated from the roots and stems of AI and SG. The novelty of the synthetic strategy is that the targeted compound was prepared by enzymatic ROP of methyl 3-(3,4-dibenzyloxyphenyl)glycidate using lipase from *C. rugosa.* It should be noted that the oligomers formed are the first enzymatically obtained analogues of the natural polyether-PDHPGA.

Antibacterial assessment of natural polyether from different species of Boraginaceae family PDHPGA-SA, PDHPGA-SC, PDHPGA-SG, PDHPGA-AI, PDHPGA-CO, and synthetic polymers PMDMPO, PMDBPO, and PMDHPO revealed that only PMDHPO showed antimicrobial activity against pathogenic strains *S.aureus* ATCC 25923 and *E.coli* ATCC 25922 at the concentrations used.

The synthetic PMDHPO would be an interesting target for diverse biological tests, and the potential enzymatic demethylation of this product could lead to a truly synthetic analogue of PDHPGA.

## Figures and Tables

**Table 1:** Antibacterial activity reported in terms of MIC against bacterial strains grown as planktonic cell cultivation on MHB.

Strains	PMDHPO MIC (µg/mL)
*S. aureus* ATCC 25923	100
*E. coli* ATCC 25922	100
*P. aeruginosa* ATCC 15442	No activity up to 100
*E. faecalis* ATCC 29212	No activity up to 100

## Data Availability

All data obtained by this study are included in this article.
